# Epidemiology of Vector-Borne Pathogens Among U.S. Government Working Dogs

**DOI:** 10.1089/vbz.2020.2725

**Published:** 2021-04-27

**Authors:** Alyssa C. Meyers, Lisa Auckland, Hannah F. Meyers, Carlos A. Rodriguez, Eric Kontowicz, Christine A. Petersen, Bruno L. Travi, John P. Sanders, Sarah A. Hamer

**Affiliations:** ^1^ Department of Veterinary Integrative Biosciences, College of Veterinary Medicine and Biomedical Sciences, Texas A&M University, College Station, Texas, USA.; ^2^ Department of Chemistry, Kalamazoo College, Kalamazoo, Michigan, USA.; ^3^ Texas A&M Veterinary Medical Diagnostic Laboratory, College Station, Texas, USA.; ^4^ Department of Epidemiology, College of Public Health, University of Iowa, Iowa City, Iowa, USA.; ^5^ Department of Internal Medicine (Infectious Diseases) and Microbiology and Immunology, University of Texas Medical Branch, Galveston, Texas, USA.; ^6^ Office of Workforce Health and Safety, Department of Homeland Security, Office of the Chief Human Capital Officer, Washington, District of Columbia, USA.

**Keywords:** *Trypanosoma cruzi*, *Dirofilaria immitis*, *Ehrlichia* spp, *Anaplasma* spp, *Borrelia burgdorferi*, *Leishmania* spp

## Abstract

Surveillance of U.S. domestic dogs for exposure to vector-borne pathogens can identify regions of transmission that are relevant for human and animal health. Working dogs with high levels of outdoor exposure may be sensitive indicators of local risk, owing to increased contact with vectors. We randomly selected 476 high-value government working dogs from 40 states to determine the prevalence of infection with *Dirofilaria immitis* and *Rickettsia* spp., and exposure to *Ehrlichia* spp., *Anaplasma* spp., and *Borrelia burgdorferi*, and identify risk factors for positivity. Additionally, we tested 100 of these dogs from Texas for *Leishmania* spp. where sand fly vectors occur. Previously published *Trypanosoma cruzi* infection data on these dogs were used to identify coinfection or co-exposures. Infection prevalence was 0.84% for *D. immitis*, and all dogs were negative for *Rickettsia* spp. DNA. Seroprevalence of each pathogen was: *B. burgdorferi* 0.84%, *Ehrlichia* spp. 1.3%, *Anaplasma* spp. 1.5%, *Leishmania* spp. 2.0%, and *T. cruzi* 12.2%. Coinfection or co-exposure took place in four (0.84%) dogs. In bivariable analysis, we found that *D. immitis*-positive and *Ehrlichia*-seropositive dogs were significantly older than negative dogs (*p* < 0.05). Furthermore, seroprevalence of *Anaplasma* spp. was significantly higher among dogs in the Northeast United States relative to other areas of the country (4.7% vs. ≤1.4%; *p* = 0.041). Although autochthonous *Leishmania* infections have been described in the United States, the cases reported herein may represent imported *Leishmania* infection. Most federal working dogs are bred in Europe, where the parasite is endemic and congenitally transmitted. Serological cross-reaction between *T. cruzi* and *Leishmania* spp. complicates diagnosis. In this study, the use of multiple testing strategies in a comparative complementary manner provided evidence for these dogs' true exposures. Comprehensive surveillance for vector-borne pathogens in dogs can improve clinician awareness and target prevention and treatment in a One Health manner.

## Introduction

Domestic dogs serve as reservoirs, sentinels, and physical transporters for multiple zoonotic vector-borne disease (VBD) systems, with the potential to maintain vectors and pathogens in domestic environments (Fritz 2009, Otranto et al. 2009a, Day 2011). VBDs are caused by a diversity of pathogens, including protozoa, helminths, viruses, and bacteria, and can be transmitted by a range of arthropods, such as ticks, triatomines, fleas, phlebotomine sand flies, and mosquitoes. Exposure to vector-borne pathogens can be highly focal over space and time, and regions of risk may fluctuate as ideal conditions or reservoir host populations expand or decline (Hamer et al. 2008, González et al. 2010, Levi et al. 2012, Levy et al. 2014, Mahachi et al. 2020). Accordingly, epidemiological data on vector-borne infections in dogs can be useful in identifying areas of risk.

Throughout the United States, multiple vector-borne pathogens are of concern in dogs and many are zoonotic. *Dirofilaria immitis*, causative agent of heartworm disease, is vectored by mosquitoes in the genera *Aedes*, *Anopheles*, and *Culex* (Ledesma and Harrington 2011, Dantas-Torres and Otranto 2013). *Borrelia burgdorferi* and *Anaplasma phagocytophilum* are primarily vectored by *Ixodes* spp. ticks, sharing a geographic distribution and seasonality (Fritz 2009). *Ehrlichia* spp. is primarily transmitted by the brown dog tick, *Rhipicephalus sanguineus* (Harrus et al. 1999). The Rickettsiaceae family causes several diseases of human and veterinary concern, including Rocky Mountain spotted fever (RMSF), caused by *Rickettsia rickettsii*. Vectors of *R. rickettsii* include *Dermacentor variabilis*, *Dermacentor andersoni*, and *Rh. sanguineus*, with infections most common in south-central and southeastern states (Demma et al. 2006a, 2006b, Fritz 2009, Nicholson et al. 2010, Foley et al. 2019). Finally, the protozoan parasites *Trypanosoma cruzi* and *Leishmania* spp. are vectored by triatomine insects and phlebotomine sand flies from the genus *Lutzomyia*, respectively (Bern et al. 2011, Esch and Petersen 2013).

Heartworm, tick, and flea control are often a part of regular preventative care in dogs, whereas testing for *B. burgdorferi*, *Ehrlichia* spp., and *Anaplasma* spp., is frequently performed in conjunction with annual heartworm testing. However, other zoonotic pathogens such as *T. cruzi*, *Leishmania* spp., and *Rickettsia* spp. are not regularly tested for or diagnosed. Infection with these vector-borne pathogens can range from subclinical infections to fatal illness (Neer et al. 2002, Chapman et al. 2006, McCall et al. 2008, Barr 2009, Mazepa et al. 2010, Day 2011, Simón et al. 2012). Some of these VBDs have long incubation times making them difficult to diagnose and treat (Straubinger et al. 1998, Dantas-Torres et al. 2006, Allison and Little 2013, Gürtler and Cardinal 2015). Thus, dogs can harbor undetected pathogens with zoonotic potential, which could have public health implications (Gürtler et al. 1986, Mather et al. 1994, Shaw et al. 2001, Dantas-Torres 2007, Lee et al. 2010).

In some zoonotic vector-pathogen systems, domestic dogs have been identified to serve as sentinels, thereby providing an indication of the relative risk of human infection with vector-borne pathogens in the same geographic area. For example, in South America, dogs are important reservoirs and sentinels of *T. cruzi* and have been used as a model to better understand the pathogenesis of *T. cruzi* infection (Castañera et al. 1998, Gürtler and Cardinal 2015). In endemic areas, dogs are a dominant reservoir host for *Leishmania infantum* bridging sylvatic to domestic transmission (Gramiccia and Gradoni 2005, Petersen 2009). The range of *B. burgdorferi* has been expanding across the United States (Eisen et al. 2015, Schwartz et al. 2017). Tracking exposure in dogs could allow for early detection of geographic expansion of endemic areas or help recognize hyperendemic foci, therefore allowing targeted intervention for humans and dogs (Lindenmayer et al. 1991, Duncan et al. 2004). In addition, dogs have been used as sentinels for human infection with *R. rickettsii* and facilitated the identification of locally infected people (Joseph et al. 2002, Elchos and Goddard 2003).

Our objectives were to determine the levels of infection and exposure to several vector-borne pathogens among working dogs across the United States that have high outdoor exposure and work and live in close proximity to humans. Additionally, we identified demographic and geographic risk factors associated with vector-borne infections and exposures and further determined whether co-exposures or coinfections occur more or less commonly than expected. Surveillance of vector-borne pathogens in dogs can allow for targeted prevention, diagnosis, and treatment and can inform both public and veterinary health programs.

## Materials and Methods

### Ethics statement

All canine samples were collected in adherence with animal use protocols approved by Texas A&M University's Institutional Animal Care and Use Committee under the numbers 2015-0289 and 2018-0460. Written consent was received for each dog sampled from their handler.

### Study population: Department of Homeland Security working dogs

The U.S. Department of Homeland Security (DHS) owns more than 3000 working dogs deployed across the United States and assigned to various task forces, including Federal Protective Services, U.S. Coast Guard, Secret Service, Transportation Security Administration (TSA), Border Patrol, and Port of Entry. The majority of these dogs are bred and purchased from Europe, but a few are from vendors across the United States. Dogs receive ∼3–6 months of training at one of four training facilities including Virginia, Alabama, and two in Texas. Dogs specialize in different jobs and are assigned to a task force and management area after which they have limited travel (with the exception of Secret Service dogs, which travel both within and outside the country). When dogs are off duty, they are either kenneled individually at their handler's residence or in a group kennel, which can be indoors or outdoors.

### Sample collection and selection

Blood samples were initially collected between 2015 and 2018 across 41 states for detailed investigations of *T. cruzi* infections (Meyers et al. 2017, 2020b). Sampling criteria included DHS working dogs over 6 months of age and on active duty. Demographic information was collected including age, sex, breed, canine job, sleeping location (home or kennel, indoors/outdoors), station of duty, and address. All dogs were assigned a unique identification number, and data were compiled into a master database with samples stored in −80°C.

From this database of more than 1600 dogs, we used a geographically stratified random process to select 476 dogs since working dogs are not evenly distributed across the United States and are concentrated in border regions or large cities. Latitude and longitude lines at 5° increments were used to stratify the continental United States. From each grid square, either all available dogs were selected or when large sample sizes were available, dogs were randomly selected using a random number generator. The sample set of 476 was subjected to all tests described below with the exception of *Leishmania* spp. testing, where a random subset of 100 dogs from Texas where sand fly vectors occur were selected (McHugh et al. 1993, González et al. 2010).

### Serology

Multiple independent assays were used to test dog serum or blood for antibodies to vector-borne pathogens. First, we used a commercially available rapid format enzyme-linked immunosorbent assay, the SNAP^®^ 4Dx^®^ Plus (IDEXX, Westbrook, ME), for detection of antigen to *D. immitis* (heartworm) and antibodies to *B. burgdorferi*, *Ehrlichia canis*, *Ehrlichia ewingii*, *A. phagocytophilum*, and *Anaplasma platys*. The SNAP 4Dx Plus diagnostic test kit is used widely across the United States in veterinary clinics and has been shown to have high sensitivity and specificity when compared with the diagnostic gold standard for each pathogen (Bowman et al. 2009).

To detect visceral *Leishmania* spp. in the subset of 100 dogs from Texas, we used the Kalazar Detect™ Rapid Test Canine (InBios International, Inc., Seattle, WA), which is a rapid immunochromatographic strip assay utilizing recombinant antigen K39 for the qualitative detection of antibodies to *Leishmania* spp. in dogs (Da Costa et al. 2003). The K39 antigen is a highly conserved repetitive immunodominant epitope in some *Leishmania* spp., including *Leishmania donovani* and *L. infantum* (Burns et al. 1993). If a dog was positive and when available, samples from early or later time points were also evaluated on the same testing platform to determine the apparent duration of seropositivity (Meyers et al. 2017, 2020a, 2020b).

Positive samples as determined by Kalazar Detect Rapid Test Canine plus 15% of the negatives were tested by indirect fluorescent antibody (IFA) for the detection of antibodies to *L. infantum* run by the Texas A&M Veterinary Medical Diagnostic Laboratory (TVMDL, College Station, TX). Since whole-cell IFAs cross-react between *L. infantum* and *T. cruzi*, any sample that reacted on the *L. infantum* IFA was then re-run in parallel with the *T. cruzi* IFA using the same dilution series and interpreted by the same diagnostician to reduce subjectivity and day-to-day variation. Samples with endpoint titer values of ≥20 were considered positive.

### Molecular detection

DNA was extracted from ∼250 μL of buffy coat using a commercial spin-column based kit (E.Z.N.A. Tissue DNA kit; Omega Bio-Tek, Norcross, GA). For *Rickettsia*, all samples were screened by PCR using primers RrCS 372 and RrCS 989 to amplify a 617 bp segment of the citrate synthase gene (gltA) to detect spotted fever group *Rickettsia* (Kollars and Kengluecha 2001, Williamson et al. 2010). Each reaction contained 1.5 μL of extracted DNA, 0.67 μM each of RrCS-F and RrCS-R primers (Integrated DNA Technologies, Coralville, IA), and PreMix E with FailSafe PCR Enzyme (Epicentre, Madison, WI) in a final volume of 15 μL. Positive and negative PCR controls consisted of *Rickettsia parkeri* DNA extracted from a tick in Texas (Castellanos et al. 2016) and water, respectively. Amplicons were visualized on 1.5% agarose gels, and samples that yielded a band of the appropriate size were cleaned using ExoSAP-IT PCR Product Cleanup Reagent and sent to Eton Bioscience, Inc., (San Diego, CA) for Sanger sequencing.

DNA from *B. burgdorferi* serologically positive dogs' blood was submitted to multiplex real-time (RT) PCR with probes for the 16S rDNA of Lyme group *Borrelia* and relapsing fever group *Borrelia* (Tsao et al. 2004). Reactions included 3 μL of extracted DNA, 900 nM each primers, 200 nM each probe, and iTaq University Probes Supermix (Bio-Rad Laboratories, Hercules, CA) in a final volume of 15 μL using previously described probes and primers (Tsao et al. 2004). Positive controls including *B. burgdorferi* DNA extracted from a tick from Tennessee (Hickling et al. 2018) and *Borrelia lonestari* DNA extracted from a field-collected *Amblyomma americanum* tick from central Texas and a negative water control were incorporated into each run.

The 100 dogs that were selected for antibody testing for *Leishmania* spp. were also tested for *Leishmania* spp. DNA using by RT-PCR. Primers for kinetoplastid DNA that detect both *L. infantum* and *Leishmania mexicana*: F 5′-AAGTGCTTTCCCATCGCAACT, R 5′-GACGCACTAAAC CCCTCCAA (Integrated DNA Technologies) and TaqMan probe, 5′-6FAM-CGGTTCGGTGTGTGGCGCC-MGBNFQ (Applied Biosystems, Foster City, CA), were modified from previously published primers (Toepp et al. 2017). Primers were used at 775 nM and probe at 150 nM, with thermocycling at 50°C for 2 min, 95°C for 3 min, and 50 cycles of 95°C for 15 s, and 60°C for 1 min. Positive controls, including *L. mexicana* from a Texas cat and *L. infantum* from a fluorescent antibody substrate slide (catalogue no. SLD-IFA-LSH; Veterinary Medical Research & Development, Inc., Pullman, WA), and negative (water) controls were included in each PCR.

### Statistical methods

**FIG. 1.**
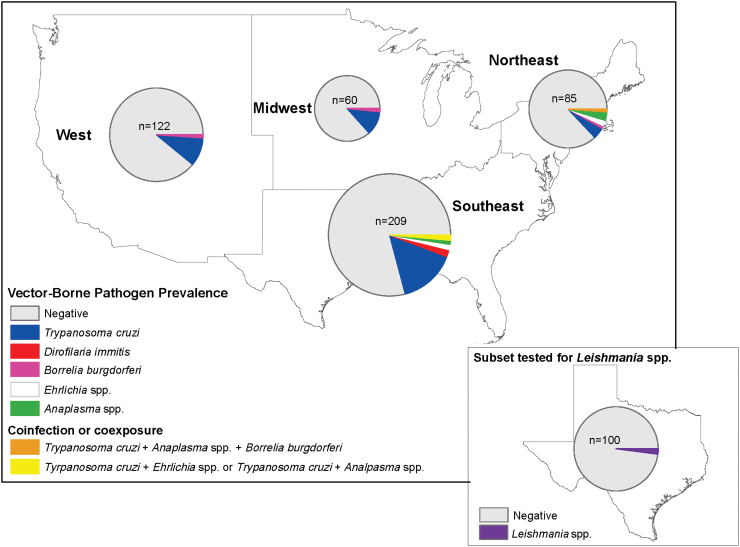
Evidence of *Dirofilaria immitis* antigen or antibodies to *Trypanosoma cruzi*, *Borrelia burgdorferi*, *Ehrlichia* spp., *Anaplasma* spp., or *Leishmania* spp. in DHS dogs across the United States. *Circles* are proportional to the sample size. Dogs were sampled from six different task forces within the DHS; all dogs were trained in the southern United States. Map was created using ArcMap 10.7.1 with a modified base layer of U.S. regions downloaded from www.census.gov and Texas layer from http://gis-txdot.opendata.arcgis.com/ DHS, Department of Homeland Security. Color images are available online.

Data were imported into Program R version 4.0.1. Bivariable analysis using chi-squared test or the Fisher exact test was performed to evaluate the relationship between the putative risk factors and infection or exposure status. Variables included: task force (Federal Protective Services, U.S. Coast Guard, Secret Service, TSA, Border Patrol, and Port of Entry), sleeping location (indoors or outdoors), sex, age, and region (Midwest, Northeast, Southeast, West; Fig. 1). Continuous factors were assessed for normality using the Shapiro–Wilk test and analyzed using the nonparametric Mann–Whitney *U*-test. *p* Values below 0.05 were considered significantly different. We used the Fisher exact test to determine if coinfections or co-exposures (defined as a sample that was positive for more than one pathogen) were more or less common than would be expected due to chance.

## Results

A total of 476 dogs were sampled across the United States; demographic data are reported in Table 1. Dogs were from 40 states, and the number of dogs sampled from each region ranged from 209 dogs in the Southeast (43.9%) to 60 dogs in the Midwest (12.6%; Table 1). The majority of dogs were male (68.7%), slept indoors (63.9%), and the mean/median age was 5.6/5.5 years (Table 1). More than half of the dogs (54.4%) were working for the TSA, with the second largest task force being Border Patrol (24.6%).

**Table 1.** Demographic Data and Results of Bivariable Analysis of Potential Risk Factors for Vector-Borne Disease in 476 Government Working Dogs Across the United States

Variable	Sample size,* n *(%)	Trypanosoma cruzi positive (%)	*p*	Dirofilaria immitis positive (%)	*p*	Borrelia burgdorferi positive (%)	*p*	Ehrlichia spp. positive (%)	*p*	Anaplasma spp. positive (%)	*p*
Sex											
Male	327 (68.7)	38 (11.6)	0.68	3 (0.92)	1.00	3 (0.92)	1.00	5 (1.5)	0.67	5 (1.5)	1.0
Female	149 (31.3)	20 (13.4)		1 (0.67)		1 (0.67)		1 (0.67)		2 (1.3)	
Task force											
Border Patrol	117 (24.6)	26 (22.2)	0.0020	4 (3.4)	0.066	0 (0)	0.21	3 (2.6)	0.16	2 (1.7)	0.58
Coast Guard	10 (2.1)	1 (10.0)		0 (0)		0 (0)		1 (10.0)		0 (0)	
Federal Protective Services	17 (3.6)	3 (17.6)		0 (0)		1 (5.9)		0 (0)		1 (5.9)	
Port of Entry	57 (12.0)	6 (10.5)		0 (0)		1 (1.8)		0 (0)		0 (0)	
Secret Service	16 (3.4)	3 (18.8)		0 (0)		0 (0)		0 (0)		0 (0)	
TSA	259 (54.4)	19 (7.3)		0 (0)		2 (0.77)		2 (0.77)		4 (1.5)	
Location											
Midwest	60 (12.6)	7 (11.7)	0.07	0 (0)	0.25	1 (1.7)	0.081	0 (0)	0.28	0 (0)	0.041
Northeast	85 (17.9)	5 (5.9)		0 (0)		2 (2.4)		2 (2.4)		4 (4.7)	
Southeast	209 (43.9)	34^a^ (16.3)		4 (1.9)		0 (0)		4 (1.9)		3 (1.4)	
West	122 (25.6)	12 (10.9)		0 (0)		1 (0.82)		0 (0)		0 (0)	
Sleeps											
Indoors	304 (63.9)	35 (11.5)	0.65	4 (1.3)	0.30	4 (1.3)	0.30	3 (1.0)	0.67	5 (1.6)	1.0
Outdoors	172 (36.1)	23 (13.4)		0 (0)		0 (0)		3 (1.7)		2 (1.2)	
Average age^b^ (negative/positive)	—	5.6/6.0	0.083	5.6/8.3	0.032	5.6/5.7	0.97	5.6/7.4	0.035	5.6/6.7	0.26
Total	476	58 (12.2)		4 (0.84)		4 (0.84)		6 (1.3)		7 (1.5)	

All dogs were tested for *Rickettsia* spp. DNA but were negative.

^a^ Thirty-five total positive dogs, one dog was PCR positive serology negative and not included in the calculation.

^b^ Mann–Whitney test performed.

TSA, Transportation Security Administration.

Of the 476 working dogs tested for exposure or infection with multiple vector-borne pathogens (excluding *Leishmania* spp.), 74 (15.5%) were reactive for at least one pathogen. The prevalence of *D. immitis* was 0.84%, and no dogs were positive for *Rickettsia* spp. as determined by PCR (Table 1). The overall seroprevalence of each pathogen was *B. burgdorferi* 0.84%, *Ehrlichia* spp. 1.3%, *Anaplasma* spp. 1.5% and *T. cruzi* 12.2%. All four dogs that were antibody positive for *B. burgdorferi* were tested for *Borrelia* DNA in the blood using PCR, and all were negative.

Of the 100 working dogs in Texas that were tested for antibodies to *Leishmania* spp., 4 dogs reacted on Kalazar Detect Rapid Test Canine. Two of these dogs were negative on all *T. cruzi* serology assays including 1 which had an IFA titer of 40 (*i.e.*, was positive at a dilution of 1:40) for *L. infantum* (dog IDs 4 and 5, Table 2). These dogs were considered positive for *Leishmania* antibodies. The remaining two dogs reactive on Kalazar Detect Rapid Test Canine were consistently positive for multiple *T. cruzi* serology assays over the years tested (2015, 2017, and 2019, dog IDs 6 and 7, Table 2) (Meyers et al. 2017, 2020a, 2020b). One of these dogs was consistently positive all 3 years on Kalazar Detect, and the other was only positive in 2019. In a conservative analysis, these two dogs were consider to have primary *T. cruzi* responses with cross-reaction to *L. infantum*, yielding 2.0% seroprevalence for *Leishmania* spp. There was no significant difference among the dogs that underwent *Leishmania* spp. testing based on sex, task force, sleeping location, or age (*p* > 0.05, Table 3). However, a more inclusive estimate encompassing coinfection or co-exposure yields a seroprevalence of 4.0%. Of the random subset of dogs that were negative by Kalazar Detect Rapid Test Canine and tested on the *Leishmania* IFA, three dogs had titers (dog IDs 1–3, Table 2). These dogs had a follow-up *T. cruzi* and *L. infantum* IFAs run in parallel, at which time two dogs had titers of 320 and 640 for *T. cruzi* and *L. infantum*, respectively, whereas the third dog had titers of 2560 for both parasites. These three dogs were positive on both *T. cruzi* rapid tests. Because *Leishmania* spp. and *T. cruzi* IFAs are known to cross-react, these samples were interpreted as a primary *T. cruzi* response with cross-reaction to *L. infantum*.

**Table 2.** *Trypanosoma cruzi* and *Leishmania* spp. Serology and PCR Results on Seven Government Working Dogs from Texas

ID	Chagas Stat-Pak^®^	Initial* Trypanosoma cruzi *IFA endpoint titer	Chagas Detect™ Plus	T. cruzi RT-PCR on blood	Initial* Leishmania infantum *IFA endpoint titer	Kalazar Detect™ Rapid Test Canine	Leishmania spp. RT-PCR on blood	Repeated side-by-side* T. cruzi/L. infantum *IFA	Diagnostic conclusion
1	Pos.	320	Pos.	Neg.	1024	Neg.	Neg.	320/640	Cross-reaction to *L. infantum* on IFA
2	Pos.	320	Pos.	Neg.	512	Neg.	Neg.	320/640	Cross-reaction to *L. infantum* on IFA
3	Pos.	1280	Pos.	Neg.	2048	Neg.	Neg.	2560/2560	Cross-reaction to *L. infantum* on IFA
4	Neg.	Neg.	Neg.	Neg.	Neg.	Pos.	Neg.	N/A	*L. infantum* (*T. cruzi* Neg.)
5	Neg.	Neg.	Neg.	Neg.	40	Pos.	Neg.	N/A	*L. infantum* (*T. cruzi* Neg.)
6	Pos.	Neg.	Pos.	Neg.	Neg.	Pos.	Neg.	N/A	*T. cruzi*
7	Neg.	40	Pos.	Neg.	Neg.	Pos.^a^	Neg.	N/A	*T. cruzi*

^a^ Kalazar Detect was negative in 2015 and 2017 but positive in 2019.

IFA, indirect fluorescent antibody; Neg., negative; Pos., positive; RT-PCR, real-time PCR.

**Table 3.** Demographic Data and Results of Bivariable Analysis of Potential Risk Factors for Infection with *Leishmania* spp. in 100 Government Working Dogs Across the United States

Variable	Sample size	Leishmania spp. positive (%)	*p*
Sex			
Male	70	2 (2.9)	1.0
Female	30	0 (0)	
Task force			
Border Patrol	76	1 (1.3)	0.42
Port of Entry	24	1 (4.2)	
Sleeps			
Indoors	43	0 (0.0)	0.50
Outdoors	57	2 (3.5)	
Average age^a^ (negative/positive)		6.1/5.3	0.59
Total	100	2 (2.0)	

^a^ Mann–Whitney test performed.

All 100 dogs tested by PCR for *Leishmania* spp. were negative.

Coinfection or co-exposure took place in four (0.84%) dogs: two with antibodies to both *Ehrlichia* spp. and *T. cruzi*; one dog with antibodies to *Anaplasma* spp. and *T. cruzi*; and one dog with antibodies to *Anaplasma* spp., *T. cruzi*, and *B. burgdorferi*. All coinfected dogs came from the Southeast, with the exception of the tri-infection dog, which was from the Northeast.

*D. immitis*-positive dogs were found exclusively in the Southeast, whereas *Ehrlichia* spp.- and *Anaplasma* spp.-positive dogs were found only in the Northeast and Southeast (Fig. 1). *B. burgdorferi*-positive dogs came from all locations, except for the Southeast. The highest percentage of *T. cruzi*-positive dogs came from the Southeast (16.3%), but the Midwest (11.7%) and West (10.9%) also had high seroprevalences. In the bivariable analysis, *T. cruzi* seroprevalence was significantly different across task force (*p* = 0.0020), where seroprevalence was highest in Border Patrol dogs (22.2%) and lowest in TSA dogs (7.3%, Table 1). *D. immitis*-positive and *Ehrlichia* spp.-seropositive dogs were significantly older (*p* < 0.05) than negative and seronegative dogs, respectively. The seroprevalence of *Anaplasma* spp. was significantly different by location (*p* = 0.041), where positive dogs came from the Northeast (4.7%) or Southeast (1.4%), but no dogs were positive for *Anaplasma* spp. in the Midwest or West. There were no other significant associations between measured variables and vector-borne infections/exposures.

## Discussion

Working dogs are exposed to multiple vector-borne pathogens, including some of zoonotic concern. Their exposure to *D. immitis*, *Anaplasma* spp., and *B. burgdorferi* was lower than the reported prevalence of 1–3 million pet dogs nationwide (Bowman et al. 2009) and lower than tick-borne pathogens in a nationwide survey of hunting dogs (Mahachi et al. 2020). In contrast, the *Ehrlichia* spp. prevalence of 1.3% in the working dogs was higher than that of *E. canis* in pet dogs (Bowman et al. 2009); our higher prevalence of *Ehrlichia* spp. could be due to the detection of antibodies specific to both *E. ewingii* and *E. canis*. In some regions, *E. ewingii* is the primary pathogen of canine ehrlichiosis; in the south-central United States, 44.8% of *Ehrlichia*-infected dogs had antibodies specific to *E. ewingii* (*n* = 143) compared with 1.4% with antibodies to *E. canis* and 17.5% to *Ehrlichia chaffeensis* (Little et al. 2010). The trend toward a slightly lower prevalence across several vector-borne pathogens in DHS working dogs is likely due to rigorous protocols for year-round, regular use of heartworm preventatives, and ectoparasiticides against arthropods in this dog population; although a specific drug might vary by region or task force, all dogs were on prophylactic drugs.

Studies exploring the prevalence of spotted fever group of rickettsiae in dogs are limited in the United States. In regions of recent outbreaks of human RMSF in northern Mexico associated with *Rh. sanguineus* ticks, no dogs were PCR-positive for *R. rickettsii* yet the canine seroprevalence was remarkably high at 65% (Foley et al. 2019). We found no dogs with rickettsial DNA in their blood, suggesting that dogs were not actively infected with *Rickettsia* spp. at the time of testing (Kidd et al. 2008). *Rickettsia* spp. can be found for about 6–10 days in blood after initial infection (Nicholson et al. 2006), circulating at very low numbers during the acute stage of infection in dogs and the sensitivity of PCR assays is low (Parola et al. 2005, Kidd et al. 2008). Further study of canine infection with *Rickettsia* spp. could be useful in evaluating the dog's role as both sentinels and reservoirs for spotted fever group of rickettsiae.

Differentiating between *Leishmania* spp. and *T. cruzi* infections presents diagnostic challenges, owing to serological cross-reactions and the amastigote stage of both parasites being identical (Williams et al. 1977, Nabity et al. 2006, Barr 2009). Furthermore, in our study, it is biologically plausible that dogs could be infected with either parasite. We interpreted test results from two dogs in our study as seropositive for *Leishmania* spp., most likely representing imported cases of *L. infantum* from the Netherlands where they were bred (Díaz-Espiñeira et al. 1997, Teske et al. 2002, Maia and Cardoso 2015). *L. mexicana* causes cutaneous leishmaniasis (CL) and is endemic to Texas (Gustafson et al. 1985, Barnes et al. 1993, McHugh et al. 1995, Wright et al. 2008, Trainor et al. 2010), but cross-reaction on Kalazar Detect Rapid Test with CL species is unlikely in both humans and dogs (Lemos et al. 2008, Molinet et al. 2013). Two additional dogs had positive rapid test results and were considered cross-reaction with *T. cruzi* antibodies, but alternatively could be coinfected with *T. cruzi* and *Leishmania* spp., which have been found in dogs, humans, and wildlife (Alcântara et al. 2018, de Oliveira Porfirio et al. 2018, Viettri et al. 2018).

For three dog samples, both the *Leishmania* spp. and *T. cruzi* IFA were positive. The *T. cruzi* IFA uses the whole epimastigote, whereas the *Leishmania* spp. IFA uses the whole promastigote form of the parasite and are known to cross-react between antibodies to *T. cruzi* and *Leishmania* spp. Based on positive *T. cruzi* rapid tests and negative *Leishmania* rapid test, these three animals were considered as seropositive for *T. cruzi* since the degree of cross-reaction on the *T. cruzi* rapid tests is thought to be low (Umezawa et al. 2003, Lemos et al. 2008, Zanette et al. 2014). Our apparent seroprevalence of 2% for *Leishmania* spp. is lower than what has been found in a retrospective study on Foxhounds in kennels in North America where a seroprevalence was 8.9% and the authors concluded that transmission was dog-to-dog (Duprey et al. 2006), a scenario that is less likely for the working dogs, which are usually housed individually at the handler's homes.

No dog blood samples were PCR-positive for *Leishmania* spp. at the time of sampling. Parasitemia in dogs with leishmaniasis is low and intermittent (Manna et al. 2008, Maia et al. 2009), and PCR on blood is often negative in a healthy asymptomatically infected dogs (Larson et al. 2017). Dogs are an important reservoir host of *L. infantum* in endemic areas (Dantas-Torres 2007) but have not been shown to play this role in the United States. In the southern United States, sand fly vectors in the genus *Lutzomyia* are present but have not been found to transmit visceral leishmaniasis (VL) species. An experimental study fed *Lutzomyia shannoni*, a species present in the southern United States, on *L. infantum*-infected dogs and demonstrated vector infection, leading the authors to conclude that locations where infected dogs and *L. shannoni* overlap, new endemic cycles of VL could be established (Travi et al. 2002). These findings have important management implications for infected dogs.

Geographically, the working dogs in our study showed the highest prevalence of *D. immitis* and *Ehrlichia* spp. in the Southeast. Previous studies in shelter dogs and rescue dogs from Texas found a much higher prevalence for *D. immitis* and *Ehrlichia* spp. (Hodo et al. 2018, Fudge et al. 2020), which could support that Texas (included in the Southeast region) is an endemic foci or could represent a study population with greater exposure to vectors and reduced prophylactic treatment.

*B. burgdorferi* prevalence was highest in the Northeast, as expected from prior nationwide study (Bowman et al. 2009, Mahachi et al. 2020), and owing in large part to vector species distributions. For *Anaplasma* spp., we found the highest prevalence in the Northeast (*p* = 0.041) similar to findings in hunting dogs (Mahachi et al. 2020). We found that the highest seroprevalence for antibodies to *T. cruzi* was in the southeastern United States, where there is a robust enzootic transmission cycle (Bern and Montgomery 2009, Bern et al. 2011), and the detected exposures in dogs outside this range likely occurred predeployment while training in the south (Meyers et al. 2020b).

In endemic areas where multiple vectors are present, such as triatomines, mosquitoes, ticks, and phlebotomine sand flies, coinfections can result in a complex clinical expression making diagnosis difficult (Otranto et al. 2009b). We detected coinfection/exposure in four (0.84%) dogs; three dogs were exposed to *T. cruzi* and either *Anaplasma* spp. or *Ehrlichia* spp. Unexpectedly, we found one dog was positive for *Anaplasma* spp., *T. cruzi*, and *B. burgdorferi*; this dog was likely exposed to *T. cruzi* while training in the south, before being deployed to the Northeast. Similar coinfections/exposures with the tick-borne agents have been described in both humans and dogs (Belongia 2002, Beall et al. 2008, Cruz-Chan et al. 2010, Hodo et al. 2018, Fudge et al. 2020), but the clinical importance warrants further research.

VBD can have a significant impact of the health of working dogs. In the 1960s, a severe epizootic episode of canine ehrlichiosis killed 200–300 military dogs during the Vietnam War, with major financial and security consequences (Kelch 1984). In 2009, military working dogs deployed to Iraq had to be evacuated due to cardiac symptoms from *T. cruzi* infections, therefore leaving units vulnerable without explosive detection dogs (McPhatter et al. 2012). The DHS working dogs are highly trained in a diversity of security functions and potential loss of duty from the clinical outcomes of VBD could affect the workforce and have broad security consequences. Furthermore, working dogs work and live in close proximity to their human handlers and might be sensitive sentinels for environmental hazards. A study on working dogs serving in Vietnam provided evidence of dogs as sentinels for human health hazards (Hayes et al. 1990), whereas others have looked at search and rescue dogs deployed in 9/11 for indicators of asbestos exposure and development of mesothelioma in rescue workers (Otto et al. 2004).

Limitations of this study include the absence of travel histories for each dog. Without this knowledge, the infection or exposure status of the dogs' current residence may not reflect the actual geographic risk. Also, clinical assessments were not conducted, so there are no data to evaluate outcomes of the detected infections. Finally, these government working dogs represent a limited number of large dog breeds, and there may be limitations to generalizing their level of vector-borne pathogen exposure and infection to the broader populations of privately owned dogs.

## Conclusions

We demonstrated that working dogs are broadly exposed to vector-borne pathogens and could be useful sentinels for exposure in their human handlers. Our surveillance targeted the agents of several nationally or state-level reportable diseases of humans in the United States (ehrlichiosis, anaplasmosis, leishmaniasis, Lyme disease, spotted fever rickettsiosis, and Chagas disease) (Bern et al. 2019, Centers for Disease Control and Prevention 2019). Unexpectedly, we identified at least two dogs with antibodies to VL, and while the cases were likely imported, this could have important implications for human health. In endemic areas, dogs are a reservoir hosts for human VL, and these dogs reside in locations with established sand fly vectors.

Further studies are warranted to assess the zoonotic risks to humans of imported VL in dogs. In a One Health approach, surveillance studies in dogs can be informative for both veterinary and human medicine, particularly when new areas of endemicity are identified as it can inform clinicians of local risk (Duncan et al. 2004, Demma et al. 2006b).
